# Recent Advances of Processing and Detection Techniques on Crustacean Allergens: A Review

**DOI:** 10.3390/foods14020285

**Published:** 2025-01-16

**Authors:** Xin Qu, Zekun Ma, Xuli Wu, Liangtao Lv

**Affiliations:** 1 College of Food Science and Engineering, Qingdao Agricultural University, Qingdao 266109, China; 19560732072@163.com; 2 Qingdao Municipal Center for Disease Control & Prevention, 175 Shandong Road Shibei District, Qingdao 266033, China; 1989_quxin@163.com; 3 School of Public Health, Health Science Center, Shenzhen University, Shenzhen 518060, China

**Keywords:** crustacean allergens, epitopes, detection, processing methods

## Abstract

Crustaceans are delicious and highly nutritional food. However, crustaceans are one of the main food allergens, causing severe public health issues. Thus, it is important to increase the knowledge on crustacean allergens and protect the health of sensitized individuals. This review systematically summarizes the basic information on major crustacean allergens’ characteristics, structures, and function. It also summarizes the latest evaluation and detection methods of crustacean allergens. In addition, various processing techniques to alleviate crustacean’s allergenicity are discussed and compared. A host of multiplex approaches as innovative research is attractive to decrease crustacean allergenicity. In addition, the strategies to address the risk of crustacean allergens are also reviewed and discussed in detail. This review provides updates and new findings on crustacean allergens, which helps better understand crustacean allergy and provide novel strategies for its prevention and management.

## 1. Introduction

Food allergy attracts worldwide attention as a major public health concern. Specific allergenic food proteins could serve as a trigger to abnormal immune system response. Allergic symptoms generally involve mucocutaneous, anaphylactic, gastrointestinal, cardiovascular, and respiratory disorders [1]. According to the extensive and long-term epidemiological research by the World Allergy Organization, a systematic analysis was carried out on food allergy cases in multiple regions around the world. So far, 90% of food allergic incidences are believed to be caused by nine common foods including crustaceans, fish, milk, egg, peanut, soybean, nut, sesame, and wheat [2].

**Figure 1:**
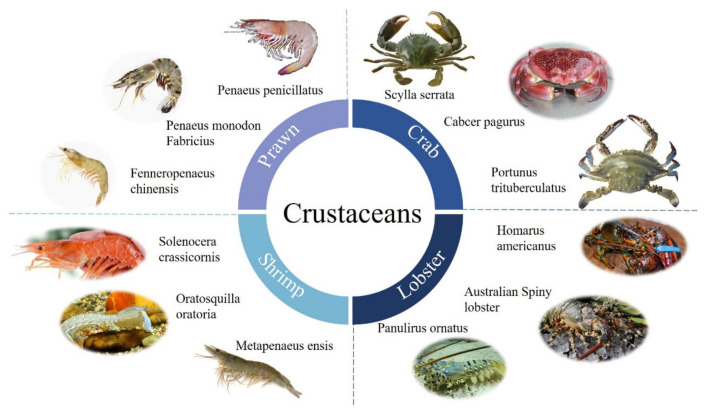
A schematic presentation of common crustaceans.

Crustaceans belong to arthropods, containing more than 50,000 species. Figure 1 presents the common crustaceans, namely crabs, lobsters, shrimp, and prawns. Generally, it is acknowledged that crustaceans are a very popular food due to their high nutritional value with high-quality proteins. However, it is also one of the major food sources capable of inducing allergic reactions. The main cause of crustacean allergy is the reaction to the various allergenic proteins in crustaceans, which include arginine kinase (AK), myosin light chain (MLC), troponin C (TnC), tropomyosin (TM), sarcoplasmic calcium-binding protein (SCP), hemocyanin, and several novel potential allergens. The allergens are primarily glycoprotein with 10–70 kDa, which contains allergic epitopes [3].

Allergenicity is primarily determined by allergic epitopes, which are classified as linear and conformational based on the continuity of the amino acid sequence [4]. The structure and sequence of crustacean allergens are similar, especially antigenic epitopes, which are the foundation of cross-reactivity between diverse crustaceans and other species. In most cases, avoiding the intake of crustaceans is the most common management strategy for allergic individuals. Crustaceans sometimes are implemented as food additives, likely causing accidental consumption by crustacean-allergic patients. Due to the challenge of avoiding such hidden allergens, crustaceans pose a high potential threat to human health. Therefore, it is needed to develop alternative procedures like effective methods for detecting or reducing protein allergenicity.

In this review, a comprehensive and up-to-date summarization of crustacean allergens is provided. We present the information about the different characteristics and effective evaluation of crustacean allergens, as well as detection methods and various advanced techniques to reduce the allergenicity of crustacean allergens, and provide the strategies to address the risk of crustacean allergy [5]. It is conceivable that the information will provide rationales for novel strategies to prevent and manage allergy to crustaceans.

## 2. Prevalence of Crustacean Allergy

The prevalence of crustacean allergy is around 0.5–2.5% of the general population [6]. The prevalence varies with food processing methods, degree of consumption by age, and geographical and cultural eating habits. There is also a certain percentage of adults and children in developed countries who are allergic to crustaceans (US, Australia, and European countries). In the US, crustacean allergy prevalence reaches about 1.3%, of which shrimp is the top trigger. Interestingly, compared to adults, only a few children have crustacean anaphylaxis. Crustacean allergy occurrence in children and adults is 0.1% and 2% [7]. Shrimp allergy, as the primary reason of crustacean allergy, is 10.2% in Italy, 7.0% in France, and about 5.4% in some European countries and Australia, respectively. The large variation in prevalence is primarily caused by the methodology used, i.e., questionnaires vs food challenges [8].

Though this survey did not cover some countries (such as Finland and Portugal), high shrimp allergy prevalence (4.8 to 7.0%) was obtained in Europe’ coastal countries with high consumption of crustaceans [9]. In Spain and Iceland, shrimp allergy also contributes to one of the top three allergies [10]. Therefore, in Europe, the prevalence of crustacean allergy is most likely underestimated.

Further research found that crustacean allergy tends to be more prevalent in Asian countries, owing to early exposure and high degree of consumption. Interestingly, in the Asian diet, crustaceans are likely ingested very early, with a mean age of around 7 months [3]. In Southeast Asian countries, crustacean allergy prevalence cases are about 7% in adults and children [11], 5% in children in Singapore and Philippines. According to one large multicenter research, it was found that in China, among 44,156 allergic cases from 52 cities, up to 19.97% of population was crustacean-sensitized [12]. In India, up to 19.1% of schoolchildren were sensitive to food containing IgE, among which crustacean serves as the leading offender, contributing to the highest prevalence (10.3%). In consumers and seafood processors, the increase in allergic reactions might be attributed to the increasing popularity of crustaceans. As one of the eight major food allergens, crustaceans are typically correlated to allergic reactions from minor to life-threatening.

## 3. Crustacean Allergens and Their Properties

**Figure 2:**
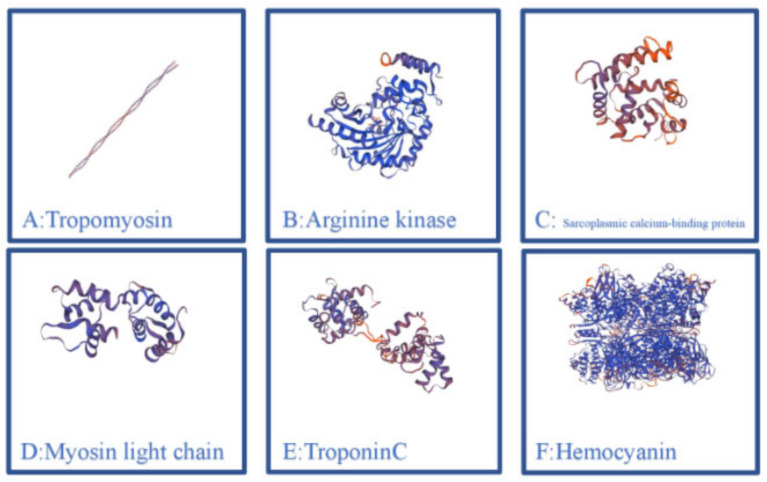
Three-dimensional structural models based on existing crystal structures (https://swissmodel.expasy.org/) for major crustacean allergens.

According to the aforementioned discussion, crustacean allergy has become a pressing global challenge that affects public health. Crustacean allergy is related to various proteins, which primarily exist in meat. Due to advanced allergen characterization and protein identification techniques, several crustacean allergens are identified. Major crustacean allergens include arginine kinase (AK), myosin light chain (MLC), troponin C, tropomyosin (TM), sarcoplasmic calcium-binding protein (SCP), and hemocyanin. Detailed information on the molecular weight, heat resistance, and functions of crustacean allergens are described in Table 1. Three-dimensional structural models in terms of existing crystal structures (https://swissmodel.expasy.org/, accessed on 6 March 2024) for major crustacean allergens are listed in Figure 2.

### 3.1. Tropomyosin

As the main invertebrate pan-allergen, tropomyosin (TM) is widely found in all edible crustaceans and registered as an allergen by World Health Organization/International Union of Immunological Societies in 13 diverse species [22]. TM-specific IgE quantification in crustacean-allergic patients revealed that most patients displayed positive binding to purified TM [23]. TM belongs to the actin filamentous binding protein family, which has different subtypes and is found in non-muscle and muscle tissues [23].It was also considered as a kind of salt-soluble acidic protein with low isoelectric point. Shrimp TM usually presents a superhelix structure composed of two α-helix subunits entwined together. Shrimp TM α-helix structure was easy to disassemble when heated at 80 °C, but it can restore its natural circular dichroism and retain its antigenicity when cooled at 25 °C [24]. In addition, TM is considered as the primary cross-reactive crustacean allergen because of the high amino acid sequence homology among species [14,23]. The high sequence homology may also be the molecular basis of cross-reactions between crustaceans, insects, and mollusks [3]. Therefore, TM is an ideal model allergen to be studied for understanding crustacean allergy.

### 3.2. Arginine Kinase

Arginine kinase (AK), with a molecular weight of 38–45 kDa, was characterized as a clinically relevant allergen in crustaceans after TM. Like TM, it was revealed that the AK has the diagnostic capability for crustacean allergy, with 10–51% of crustacean-allergic patients with positive IgE binding [22,25]. AK was detected in various crustacean species, covering black tiger prawn (*P. monodon*) [25], Pacific white shrimp (*Litopenaeus vannamei*), *Chinese shrimp* (Yao et al., 2005), and other shrimp species [26,27]. It was regarded as a monomeric phosphagen ATP phosphor transferase, which was mainly expressed in invertebrates [20]. It is likely that AK can elicit serious hypersensitivity responses and be involved in cross-reactions between mollusks and crustacean species. Moreover, AK is thermo-labile, and the intact IgE epitopes of AK could be damaged due to thermal treatment [28,29].

### 3.3. Sarcoplasmic Calcium-Binding Protein

Sarcoplasmic calcium-binding protein (SCP) is a minor allergen in crustaceans, which was identified in Pacific white-leg shrimp [30], Black tiger prawns [31], North Sea shrimp and narrow-clawed crayfish [22], and red swamp crayfish (*P. clarkii*). Only three IgE-binding epitopes were recognized in SCP compared to other crustacean allergens [16]. The IgE binding capacity of SCP reached 29–50% among shrimp-allergic patients [22]. Interestingly, IgE recognition showed a lower level in adults than in children, with a higher correlation in estimating clinical reactivity to shrimp in conjunction with TM-specific IgE. SCP is a thermo-stable protein with 20–22 kDa molecular weight containing 194 amino acids. It could be stable in acidic and alkaline conditions (pH 1–11). Moreover, as it is derived from the EF-hand calcium-binding protein family, SCP contains helix–loop–helix motifs which is dedicated to binding calcium (Ca+). The structure of shrimp SCP is composed of a dimer of polypeptide chains. The dimer contains three calcium-binding sites. SCP could influence cellular functions by controlling Ca^2+^ concentration in the cytosol. Moreover, SCP also promotes muscular relaxation via translocating Ca^2+^ from myofibrils to the sarcoplasmic reticulum, which could maintain intracellular Ca^2+^ [15,26]. The synthetic peptide technology combined with serological test was used to verify the simulated linear epitope, and the influence of the combination of SCP and Ca^2+^ on the protein structure and immune activity was explored. It was found that the specific antibody of SCP could cross-react with SCP in various shrimp, and it was considered that SCP and Ca^2+^ can be used as standard allergens for detection and desensitization of crustacean allergens.

### 3.4. Myosin Light Chain (MLC)

The MLC was initially recognized as a minor crustacean allergen in 2008 [32]. It consists of two classes, namely the essential and the regulatory light chains (MLC1 and MLC2). MLC1 has been identified in red swamp crayfish and North Sea shrimp [17], while MLC2 has been identified in white-leg shrimp, black tiger prawn, and American lobster (*Homarus americanus*). MLCs contribute to the formation of the large myosin macromolecular complex, which participates in the functional structure of muscle protein. The multimeric protein is composed of four light chains (∼18–20 kDa) and two heavy chains (∼200 kDa). Serving as the calmodulin-like protein family, the light chains bind Ca^2+^ via the EF-hand domain. Currently, there are still few studies on allergic epitopes of MLC. Yang et al. [32] only found that the MLC1 of crayfish contains three conformational epitope regions, which provide an important basis for further diagnosis research. Furthermore, MLCs also exhibited an essential influence on muscle contraction in which the actin–myosin complex was affected by its phosphorylation [18]. Similar to TM, MLC is a thermal-stable protein and tends to retain IgE-reactivity upon heat treatment. Currently, among crustaceans or other crustacean species, the immunological cross-reactivity of MLC is still unknown.

### 3.5. Troponin C

Troponin C is an allergen identified in north sea shrimp and black tiger prawn [17,33]. It is an EF-hand calcium-binding protein with a ~20 kDa molecular weight similar to SCP and MLC. Troponins bind to TM of the thin filament in order to mediate the striated muscle’s contraction. Troponin contains three subunits: (a) troponin C, (b) troponin I, (c) troponin T [3]. In response to Ca^2+^ efflux, Ca^2+^ is bound by troponin C, regulating activation. Moreover, troponin C is also thermo-labile. Compared to AK, TM, and SCP, troponin C exhibits lower IgE-binding capacity. Furthermore, troponin C could hinder actin–myosin interaction and bind to actin [3]. The prevalence of troponin C allergy may be underestimated. On the one hand, it is rather complicated to detect troponin C allergy. Currently, allergy detection methods mainly focus on common allergens, and the detection of troponin C allergy may not have been widely carried out.

### 3.6. Hemocyanin

Hemocyanin is identified as an allergen in giant freshwater shrimp and black tiger shrimp [19]. It is considered a thermo-stable allergen with a 72 kDa or a 75 kDa molecular weight. Hemocyanin is a hexamer composed of six highly helical heterologous subunits [21]. Moreover, hemocyanin not only has functions such as oxygen delivery, antiviral, and hemolysis, but also has a certain regulatory role in physiological processes such as osmotic pressure maintenance, immune factor expression, and energy conversion. For those individuals who are already allergic to crustaceans or mollusks, hemocyanin, as one of the important allergens among them, also has a relatively high incidence of causing allergies. Because their immune systems are already sensitive to this kind of food, hemocyanin can easily become one of the factors that trigger allergic reactions.

### 3.7. Other Allergic Proteins

In addition to the above six crustacean allergens, other allergic proteins are also identified in crustaceans. Paramyosin is considered a thermo-labile allergen with a 100 kDa molecular weight in different crustaceans [34]. Moreover, crustaceans also contain a few other types of allergens, including Aldolase A, β-Enolase, α-actin [35], triosephosphate isomerase (Cra c 8), myosin heavy chain (MHC) (Rahman et al., 2013), fatty acid-binding protein (FABP), glyceraldehyde-3-phosphate dehydrogenase (GADPH), and smooth endoplasmic reticulum Ca^2+^ ATPase (SERCA) [17,20]. The study of these allergens needs to be further explored.

## 4. Epitopes of Crustacean Allergens

The specific allergenic regions of crustacean allergens are called epitopes that bind to IgE, leading to allergic responses [4,36]. As they are located on the surface of allergens, antigenic epitopes are easily identified by IgE on mast cell surface. Since the whole protein allergenicity is closely associated with these epitopes, the systematical exploration of these epitopes is essential for understanding allergic reactions [37].

Many studies have been devoted to the identification of the epitope structure of crustacean allergens. The antigenic epitopes are typically analyzed by various techniques, mainly covering X-ray crystallography, phage display library, overlapping peptide library technology, inhibitory ELISA, peptide microarray method, bioinformatics, and molecular cloning. Among them, bioinformatics is the method that can predict epitopes, avoiding the disadvantage of low efficiency and cumbersome operation [38,39]. In recent years, with the advances of computer and medical science, information (like function annotation and sequence analysis) is easily available and has been extensively investigated. It has been verified that when bioinformatics was employed to predict the binding peptide chips for recognizing epitope biomarkers, the predication accuracy reached more than 90% [40]. Specifically, there are many different allergenic raw databases, such as AllerMatch (https://www.allermatch.org/, accessed on 6 March 2024), Allergen Database for Food Safety (https://www.food-safety.com/topics/312-allergens, accessed on 6 March 2024), Structure Database of Allergenic Proteins (http://fermi.utmb.edu/SDAP/sdap_who.html, accessed on 6 March 2024), the International Union of Immunological Societies (http://www.allergen.org/, accessed on 6 March 2024), the Allergen Database at the Central Science (https://ngdc.cncb.ac.cn/databasecommons/, accessed on 6 March 2024), AllergenOnline (http://www.allergenonline.org/, accessed on 6 March 2024), Allergome (http://allergome.org/, accessed on 6 March 2024), and Informall (http://www.foodallergens.info/, accessed on 6 March 2024). Different information about allergenic amino acid sequences, secondary and tertiary structures, and three-dimensional folding structures can be retrieved from the databases. In addition to using the internationally recognized database described above, the researchers also predict the antigen index of proteins and potential T-cell antigen-determining clusters by DNAStar software 17.6 and network online servers NetMHC. Xu et al. (2020) [37] analyzed the B-cell epitopes and allergenicity in shrimp tropomyosin by the bioinformatics method. Moreover, Xu et al. also identified the dominant T-cell epitopes of the Lit v 1 shrimp major allergen and their functional overlap with known B-cell epitopes [39]. Saetang et al. predicted cross-reactive epitopes of tropomyosin from shrimp and other arthropods that are involved in allergies [40]. The identification and characterization of allergen-associated epitopes provide a theoretical basis for the diagnosis, immunotherapy, and prevention of allergic reactions, as well as the development of reduced immunogenicity formula.

These techniques rely on the physicochemical properties of protein amino acid residues and polypeptide chains, such as secondary structure, hydrophilicity, flexibility, hydrophobicity, accessibility, and charge distribution, to predict the epitopes on the target protein. Because the above techniques have certain limitations when used alone, more than three techniques were combined to analyze in the actual operation process, and then other laboratory equipment was used for verification. Thus, the “High-Throughput and Bioinformatics Prediction—in vitro Validation” technology for the epitope study was developed [40]. Compared with the traditional overlapping peptide technology, this approach has the advantages of low cost, short time, and high throughput, which has been broadly applied for mapping the epitopes of food allergens. For example, this method was used to identify the critical amino acids and allergenic epitopes of AK and TM in shrimp [41].

In this review, we summarized the potential epitopes of TM recognized by applying the bioinformatics method. Allergenic epitopes can be recognized by antigenic receptors covering T-cell and B-cell receptors, but their recognition labels are completely different. Antigen processing cells are needed to process T-cell epitopes for their recognition, while B-cell epitopes could be directly identified without treatment as they typically present on the surface of antigens. Therefore, B-cell epitopes are more significant [37]. Based on the linkage of amino acid sequences, allergenic epitopes are mainly composed of conformational and linear epitopes. In general, conformational epitopes appear in B-cell epitopes, while linear epitopes appear in both T-cell and B-cell epitopes. As linear epitopes, T-cell epitopes are unable to bind to IgE. Generally, in terms of the primary or secondary structures of proteins, linear epitopes can be predicted [42]. However, little conformational epitopes data are available because of gastrointestinal digestion and thermal denaturation, and it is necessary to predict the conformational epitopes by integrating with the phage display library. Though the related information for localizing and verifying antigens could be provided by the results of prediction, it is necessary to conduct more investigation to further confirm prediction accuracy.

## 5. Cross-Reactivity of Crustacean Allergen

Cross-reactivity is caused by the recognition of a specific IgE previously generated to another similar allergen. Among IgE-mediated allergic reactions, cross-reactivity is very common. In addition, cross-reactivity is also common among different crustacean species, especially attributed to TM due to the high sequence homology and conservative antigen epitope region of this allergen [43]. Significantly, TM is generally a nonallergenic protein in vertebrate species, while in invertebrate species it is a major allergen, despite its high homology. It was found that there is no difference in the α-helix structures and thermal stability between TM from shrimp and chicken, but TM from chicken degraded faster in simulated gastric digestion and significant positive values of skin reactivity were only found in TM from shrimp [44]. Additionally, among various crustaceans and other species, the high structural similarity of TM was discovered, while cross-activity also occurred in different species, like cockroach and house dust mites [45]. In recent years, researchers revealed that the serum of nine shrimp-allergic patients had an immune response to the protein of 38 kDa of 13 different crustaceans and crustacean aquatic products. The protein has been verified to be TM. Zheng et al. compared the amino acid sequences among a variety of crustacean aquatic foods, including a variety of shrimp, lobsters, and crabs [46]. The results showed that the sequences between the TM of crustacean aquatic products were highly conservative, and most of the epitope regions were in the sequence conservative regions, which further explains the cross-reactivity between crustacean TM.

In addition, the similarity in the sequence and structure of TM provides the basis of cross-reactivity among diverse species. Among crustacean TMs, sequence homology is high with more than 98% [47,48]. Similarly, high sequence homology was achieved, 78−98% and 80−97%, among cockroach and crustacean TMs and among house dust mite and crustaceans TM, respectively. However, only 54−60% of the sequence homology was obtained among the vertebrate and crustacean TMs. Furthermore, the presence of cross-reactivity among TMs might contribute to the similarity of epitopes among eight shrimp TMs (Pen a 1), from which four are identical in dust mite and lobster TMs (Hom a 1) (Der f 10 and Der p 10) and four are the same as cockroach TM (Per a 7) [49]. It was demonstrated that 97.6% of cross-reactivity is for raw TMs between clam (*Ruditapes philippinarum*) and shrimp (*L. vannamei*) [50]. Among crustaceans and other invertebrates, 71% of mite-allergic patients exhibited cross-reactivity, displaying a possibility with 55% of being allergic to shrimp TM [51]. Cross-reactivity occurs frequently. The main reason might be the TMs from the mite, insects, and crustaceans, which all belong to the phylum arthropod.

Presently, research has mainly focused on TM. However, AK is another important allergen in crustaceans. It was found that the Atlantic snow crab AK [52] could react with the serum of patients with crustacean allergy, and the Eriocheir sinensis AK [53] has strong sensitization, as verified in Balb/c mice. Ayuso et al. found that Pacific octopus AK can bind to IgE in the serum of patients with crustacean allergy, thus confirming that AK has cross-reaction in mollusks and crustaceans [52]. Western blot showed that the polyclonal antibody of AK can react with AK of 17 different species of shrimps, crabs and crustaceans, which proves that it is an allergen in crustaceans.

It can be concluded that the amino acid sequence identity of arginine kinase (AK) is over 70% and highly conserved among crustaceans, mollusks, and even insects, which may trigger a strong immune cross-reaction [54]. It is a pan-allergen in crustaceans and aquatic animals, which may increase the difficulty of diagnosis, treatment, and prevention of crustacean allergy. More studies should be carried out to understand the cross-reactivity phenomena among crustaceans and other species.

## 6. Evaluation of the Allergenicity of Crustaceans

In recent years, the effects of different food processing technologies have been studied on crustacean allergens. So far, no method has been found to maintain the original quality and reduce the allergenicity of crustaceans. Moreover, different results could be achieved by similar processing techniques. The possible reason is that the existing evaluation technology of allergens cannot accurately reflect the change in allergenicity. Therefore, it is of utmost importance to develop methodologies to assess crustacean allergenicity. Evaluation methods rely on in silico analysis, serological analysis, simulated gastric digestion, animal sensitization models, and cell experiments.

### 6.1. Serological Analysis

Specific serum immunology analysis was contained in the allergenic evaluation decision tree released by World Health Organization (WHO) and Food and Agriculture Organization (FAO) in 2001. The use of human serology experiments could be performed to directly study the combination of allergens and IgE-specific human allergic sera. This is of great significance to provide the final evaluation of the potential allergenicity of food. At present, the research on the evaluation of food allergenicity through human serological experiments is becoming more and more substantial. For example, the potential IgE binding capacity of shrimp allergens could be evaluated with specific allergic reactions by serological testing [55]. The sensitization to crabs was analyzed using a serological assay by immunoblotting [56]. Serological analysis includes the radioallergosorbent test (RAST), Western blot (WB), Dot blot, and enzyme-linked immunosorbent assay (ELISA). These methods depend on the specific combination of antigen–antibody, which might generate false positive/negative results owing to the influence of food matrix and antibody recognition characteristics [57]. Therefore, the results of serological analysis need further additional experiments to confirm.

### 6.2. Simulated Gastrointestinal Digestion

Simulated gastrointestinal digestion is the process of digesting food in vitro using gastric and intestinal fluids to determine whether potential allergens are resistant to digestion. In the food allergic reaction, the allergenic protein needs to be absorbed by the gastrointestinal mucosa under the action of the digestive system to product immune reaction. Peptides generated from partially hydrolyzed allergens must contain relevant epitopes so that they can still remain allergenic after digestion. While simulating gastrointestinal digestion and absorption, some proteins can keep intact antigenic epitopes, even if their epitopes are exposed. Therefore, the main reason behind the stability and the sensitization ability of potential food allergens is that the allergens are digested by simulated gastrointestinal fluids in vitro to produce digestion-resistant fragments containing epitopes. In recent years, simulated gastric and intestinal fluid digestion experiments have been widely used as scientific evaluation methods of crustacean allergens. Lv et al. reported that shrimp TM is easily digested by trypsin but resistant to pepsin digestion. Ahmed et al. studied the influence of enzymatic crosslinking on the digestion stability of shrimp TM [4]. The result showed low stability of cross-linked TM in simulated gastrointestinal digestion. In addition, the IgG/IgE binding capacity of the digested fragment was also lowered. Simulating gastroenteric digestion has the advantages of being simple, safe, rapid and free from the ethical constraints of medical research. However, the simulated gastroenteric digestion experiment cannot fully illustrate the real situation of human digestion. Through the dynamic quantitative digestion stability experiment, it was found that there is no significant correlation between the digestion stability of protein in simulated gastric juice and the sensitization [58]. The components of the simulated digestive fluid contain inorganic salts and protease components. At present, the matrix environment of proteins has not been considered in the simulated digestion. Moreover, the models of adult and infant digestion are also different.

### 6.3. Animal Experiments

Animal experiments can simulate the occurrence of food allergic reactions and have wide application prospects objectively [59]. The study of animal model methods for food allergens has been carried out widely. A variety of animals such as dogs, young pigs, and mice are used in the studies. Rodents, rats, and mice are considered the best species for food allergy animal experiments because of their small size, short reproductive cycle, and rich immunological data. According to the research results of animal models, crustacean allergens can trigger IgE-mediated hypersensitivity, most of which are caused by TM-based proteins. The structure of AK partially expands, exposing hidden epitope, which may be the reason for increasing IgE reactivity. MLC shows high IgE reactivity with the sera of patients with crustacean allergy, and maintains its IgE binding capacity during processing. Up to now, six myosin light chains in crustaceans have been identified as allergens. Triosephosphate isomerase (TIM) is a new allergen in crustaceans, which can induce complex cross-reactions between species and increase the risk of sensitization. Ahmed et al. evaluated the immunomodulatory effects of transglutaminase and laccase/caffeic acid on reducing shrimp TM allergenicity using animal experiments [4]. It was also reported that the allergenicity of enzymatic cross-linked tropomyosin was estimated using mouse and cell models [60]. Although the studies showed promising performance for animal models in the allergenicity assay, in-depth studies are still needed to evaluate crustacean allergenicity by animal models. At present, young animals are generally used in animal sensitization tests, mainly because the intestinal digestive and immune system of mice at this stage have not been fully developed and are prone to allergic reactions, and mature individuals may have a certain tolerance to allergic substances. The research of animal models is still at the exploratory level. Establishing the standardization of animal models is conducive to improvement of the research level.

### 6.4. Cell-Based Assays

Compared with the simple analysis of allergenic protein structure and the allergic serum IgE levels, cell experiments can fully respond to the allergy state of the allergenic body. In the evaluation technology of allergen, the cell model is regarded as one of the most promising evaluation methods because of its advantages of simple operation, protection of animals, and proximity to the nature of the allergic reaction. The cytokine release assay is based on the fact that immune cells (such as mast cells, T lymphocytes, etc.) secrete a variety of cytokines when stimulated by allergens. These cytokines play an important role in the occurrence and development of allergic reactions. For example, interleukin-4 (IL-4) and interleukin-13 (IL-13) can promote B cells to produce IgE antibodies. By detecting the types and amounts of cytokines in the cell culture supernatant, the impact of allergens on cellular immune function can be evaluated. The release of cytokines and mediators in mast and basophils cells is an important criterion for expressing the immunoreactivity of allergenic proteins during in vitro cellular assays. The IgE-mediated KU812 and RBL-2H3 cells are reliable and commonly used models to investigate type I allergic responses using human and rat sera, individually. These assays are commonly hired to reveal the variations in IgE binding and their contingency to clinical sensitization. The magnitudes of allergic symptoms are associated with the secretion of cellular components; hence, their determinations are more valuable for scrupulously examining the state of the body in an allergic state [61]. Several researchers have found that upon treatment of allergens using different processing, the capability of the allergenic proteins to produce cytokines and mediators was significantly inhibited [46,62].

### 6.5. Oral Food Challenge

The Oral Food Challenge (OFC) is currently the “gold standard” for diagnosing food allergies. Under medical supervision, patients are gradually given the suspected allergenic foods to ingest, starting with a very small dose, and then it is observed whether the patients have allergic reactions. This method can directly determine whether the food causes allergic reactions and the severity of the reactions. The advantages of the Oral Food Challenge are as follows. Firstly, it has high accuracy. It directly observes the reactions after food intake and is the most reliable method for determining food allergies. Compared with other indirect assessment methods (such as serological tests which only detect antibody levels), it can truly reflect the situation of food-induced allergies in the body. Moreover, a comprehensive assessment of the reaction degree enables a detailed understanding of the types of allergic reactions (such as reactions in different systems like the skin, gastrointestinal tract, and respiratory tract) and their severity, which is of great importance for formulating personalized allergy management plans. For example, for patients with crustacean allergies, through the OFC, it can be determined whether it is a mild skin itch or a severe allergic reaction that may endanger life, thus guiding patients on how to avoid risks in daily life.

Overall, a technical platform for evaluating crustacean allergens in vitro and in vivo needs to be constructed with a combination of different evaluation methods. In the study of Liu et al. (2017) [59], the allergenicity of enzymatically cross-linked tropomyosin was evaluated using cell and mouse models. It was also found that the decreased allergenicity of shrimp tropomyosin with the treatment of 2,2′-azobis (2-amidinopropane) dihydrochloride (AAPH) was assayed by simulated gastrointestinal digestion, cells, and mouse models. The allergenicity of crustacean allergens was evaluated from different perspectives, which can better elucidate the changes in allergic reactions.

## 7. Detection of Crustacean Allergen

Detection of crustacean allergens is crucial for safeguarding allergic-prone individuals from inadvertent exposure to allergens. The sensitive, selective, reliable, rapid, and cost-effective analytical tools are of utmost importance for detecting trace quantities of allergens in the food process [61]. The detection methods of crustacean allergens mainly include capillary electrophoresis (CE), lateral flow immunoassay (LFIA), enzyme-linked immunosorbent assay (ELISA), polymerase chain reaction (PCR), liquid chromatography-mass spectrometry (LC-MS/MS), and biosensor technology.

### 7.1. Capillary Electrophoresis

CE is performed under a high-voltage field with a capillary as a separation lane, and the allergen is separated according to the different migration times of the sample in the buffer solution. CE has been widely used in food allergen detection fields due to its advantages of multiple separation modes, high resolution, and detection time [62]. The detection methods of CE mainly include immunoaffinity capillary electrophoresis (IACE), capillary gel electrophoresis (CGE), capillary zone electrophoresis (CZE), dynamic coating capillary electrophoresis (DCCE), and Chip capillary electrophoresis (CHip-CE). Fu et al. detected arginine kinase in crustaceans by CE. Moreover, CE also has some disadvantages, such as affecting the reproducibility of migration time, small capillary diameter, small sample volume, and great pH influence, which greatly reduces the accuracy and detection sensitivity [43]. In general, CE has various separation modes, lesser sample consumption and high separation efficiency, so it has become an inevitable trend to replace some classical electrophoresis experiments [63].

### 7.2. Enzyme-Linked Immunosorbent Assay

ELISA has been widely utilized to analyze and detect shrimp allergens [64]. It is considered a better choice for routine screening of allergens. Yu et al. detected crustacean TM in processed food by an ELISA method, with a 6.8 ng/mL detection limit and a quantitation limit of 13.67 ng/mL [65]. Seiki et al. (2007) explored a sandwich ELISA to detect and quantify crustacean allergens, which shows high specificity with minor cross-reactivities to other mollusks [66]. The allergenicity of shrimp protein extracts after Gamma irradiation was determined by ELISA. The result showed that the allergenicity of irradiated shrimp protein extracts was reduced with increasing irradiation dose. Polyclonal antibodies are relatively easy to prepare. They are usually obtained by immunizing animals (such as rabbits, goats, etc.) and then collecting their sera. Polyclonal antibodies may have broader reactivity, thus enhancing the sensitivity of detection. For example, when detecting shrimp allergens, if there are multiple isomers of the allergen or different antigenic epitopes are exposed after processing, polyclonal antibodies are more likely to recognize these changes, reducing the possibility of missed detection. Recombinant antibodies can be customized through genetic engineering techniques, and the specificity, affinity, and stability of the antibodies can be designed according to needs. The ELISA method has certain significance for food safety detection; however, the destruction of the primary structure of a sensitized protein can easily causes false-negative results in the detection of deeply processed food. Moreover, it has high selectivity to reagents, and it is impossible to analyze multiple components at one time, which causes tendency for cross-reactions.

### 7.3. Lateral Flow Immunoassay

**Figure 3:**
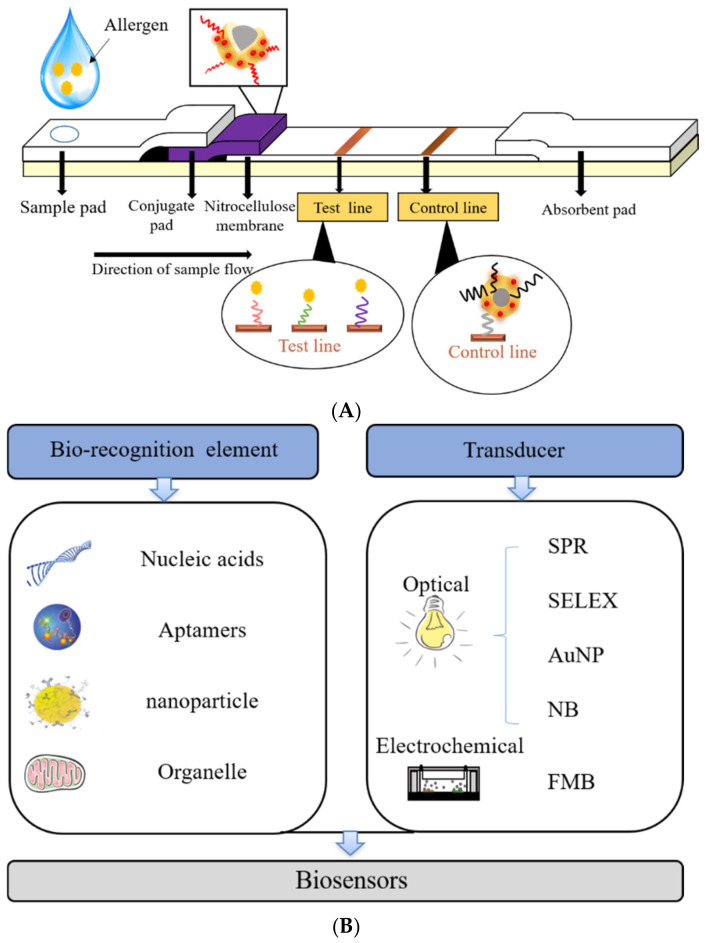
The detection of crustacean allergens by enzyme-linked immunosorbent assay. (**A**) Biosensor technology; (**B**) SPR: Surface plasmon resonance; SELEX: Systematic evolution of ligands by exponential enrichment; AuNP: Gold nanoparticle; NB: Nanomagnetic bead; FMB: Fluorescent magnetic bead.

LFIA is a commonly used immunoassay technique in recent years. The solution of the measured substance is considered as a mobile phase. Under the action of the capillary, the solution of the substance to be measured moves through the sample pad towards the absorption pad. Afterward, it first dissolves the detection antibody on the binding pad, then reacts with it, and then reacts with the antigen or antibody fixed on the quality control line and detection line. Finally, the test result is judged by the color of the quality control line and the detection line [67]. The detection principle of crustacean allergens by LFIA is shown in Figure 3A. Wang et al. detected crustacean TM based on the quantum dots-LFIA method [68]. The result could be determined within 30 min, with a 0.05 μg/mL detection limit for instrument-monitored fluorescence and a 0.5 μg/mL visual detection limit for the naked eye. LFIA shows good specificity and repeatability, but it requires accurate sample addition and is difficult to quantify. Therefore, it is not suitable for sensitive fields. Improper operation produces false-negative or false-positive results, which limits its wide application. Special marking methods are needed to overcome the limitation.

### 7.4. Liquid Chromatography–Mass Spectrometry

LC-MS/MS has gained special attention in response to the constant demand for the effective detection, quantification, and characterization of allergens [69]. This method allows the analysis of multiple food allergens in a single run with high sensitivity, specificity, accuracy, and reproducibility. Additionally, it can reduce cross-reactivity issues, often observed with immunoassay methods, by directly identifying the proteins or peptides without the interaction of allergens or marker proteins and antibodies. Moreover, this method is less influenced by food processing and food matrices, thereby allowing superior food allergen quantification [70,71]. Although liquid chromatography–mass spectrometry (LCMS) has good sensitivity, it is generally not a quantitative method for the determination of allergenic peptides. Nagai et al. developed an LC-MS/MS analysis approach to identify shrimp allergens [72]. Lv et al. applied the LC-MS/MS method to a purified shrimp allergen and an oxidized allergen, respectively [73]. The result showed that the shrimp allergen was modified by 4-hydroxy-2-nominal treatment, which reduced its allergenicity. Thereby, many studies on LC-MS/MS methods investigate the assay of crustacean allergenicity. However, LC-MS/MS methods are limited because of the problems of specificity and different cross-reactivity of antibodies.

### 7.5. Polymerase Chain Reaction

PCR, an amplification approach, is based on in vitro replication of DNA double strands. This method has been confirmed as a rapid, sensitive, and reliable approach for food authentication and proposed for various allergenic commodities. It can withstand harsh food processing conditions because of the high stability of DNA [70,74]. In the process of PCR, a primer selectively attaches to a complimentary strand of DNA which is then extended and amplified by the Taq polymerase enzyme. Agarose gel electrophoresis is used to separate the resultant product, followed by staining with a fluorescent dye, and finally, detection using UV light is performed [66]. The types of PCR for quantifying allergens contain traditional PCR, real-time PCR, and PCR integrated with ELISA, either developed in house or commercially. A real-time PCR approach to detect the lobster allergens has been recently developed. A fast real-time PCR was reported in [75] to detect crustacean allergens in foods. The method is characterized by short analysis time, high specificity, and high sensitivity. This approach has a potential to address challenging related to food safety and product labeling to ensure the allergic consumer health. However, PCR cannot directly detect allergen proteins. What PCR detects is DNA, not the allergen proteins themselves. It only detects the food containing the allergen [76].

### 7.6. Biosensor Technology

Biosensor technology was considered to utilize the selective identification and determination of biologically active substances to achieve measurement. It is widely used because of its fast, accurate, easy-to-operate, time-saving, and labor-saving characteristics, and it is convenient for computers to collect and organize data without harming samples. The methods of biosensor technology are listed in Figure 3B. Common biosensors include surface plasmon resonance (SPR), adaptor biosensors, gold nanoparticle (AuNP) biosensors, and nanomagnetic bead biosensors.

SPR is a method based on optical phenomena. The vanishing wave generated by total reflection resonates with the plasma wave in the medium, which weakens the reflected light energy and changes the refraction index, thus quantifying the protein. Marsh [45] used a surface plasmon resonance sensor and gold biochip to detect TM quantitatively, which can eliminate matrix interference of complex crustacean samples. The method causes no damage to biomolecules, does not require any markers, and has a fast response speed. However, it is sensitive to interference factors such as sample composition and temperature. Therefore, it is difficult to distinguish nonspecific substances. An adaptor biosensor is a short oligonucleotide sequence or short polypeptide obtained by in vitro screening which can specifically bind to the corresponding ligand in the sensor, with high affinity and specificity. However, the measurement accuracy is greatly influenced by light and environment. AuNP is a kind of nanomaterial with a diameter of 1–100 nm which exhibits different colors depending on its size. Wang et al. developed a biosensor based on gold nanoparticles, which are mainly used for the crustacean allergen TM [68]. This biosensor has strong specificity and accuracy, and the sample preparation is simple, which has good applicability in the detection of crustacean-related products and other food allergens. However, gold nanoparticles are not modified, which leads to poor signal fluctuation. In addition, nanometer magnetic beads also are widely used in the development of sensor technology, with their high sensitivity, reduced detection limits, and simple operation. Biosensor technology is expected to become a high-throughput detection method for food samples in the future.

In summary, all kinds of detection methods have their own unique advantages and disadvantages. At present, for the detection of crustacean allergens, there is no single method that can combine the advantages of various technologies to achieve accurate quantification and efficient analysis.

## 8. Method for Reducing the Allergenicity of Crustacean Allergens

**Figure 4:**
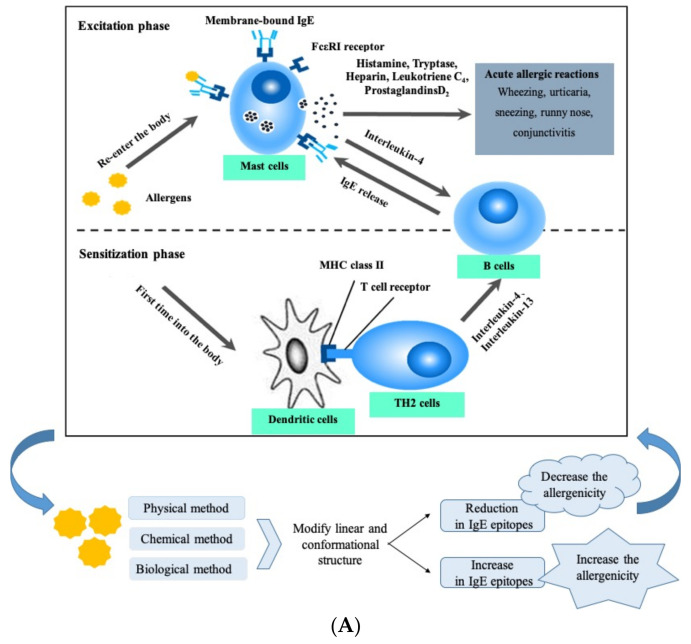

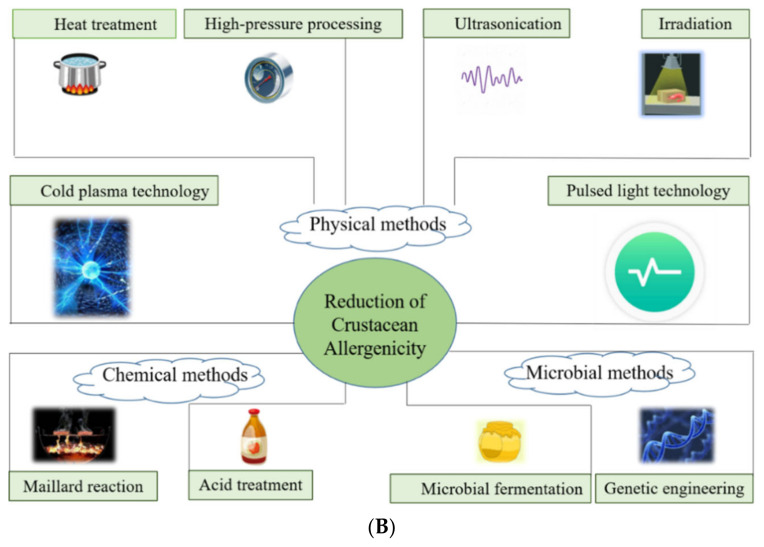
(**A**) The mechanism of food allergy and the changes in the allergenicity by different methods on crustacean allergens; (**B**) Classification of reducing crustacean allergenicity using physical, chemical, and microbial methods.

Crustacean allergy could cause severe allergic symptoms, leading to an important food safety challenge. Reducing the allergenicity of crustaceans and crustacean products has become an important approach to address food allergy risk. Figure 4A shows the changes in the linear and conformational structure by different methods and their impact on the allergenicity of crustacean allergens. An overview of the classification of the treatments is shown in Figure 4B, which may influence the markers responsible for the allergic reactions in crustaceans. A variety of methods were introduced to decrease the allergenicity of crustacean-containing foods and reduce the incidence of crustacean allergy (Table 2).

### 8.1. Physical Methods

#### 8.1.1. Heat Treatment

Heat treatment technology is applied in various ways, including water boiling, hot stream, roasting, autoclaving, frying, and so on [77,89]. Via interaction reaction, heat treatment could result in modification or attenuation of shrimp allergens. The epitopes of allergens could be damaged by heat treatment, which influences the allergenicity of allergens [90]. Epitopes are divided into linear epitopes and conformational epitopes. A linear epitope is composed of a consecutive sequence of amino acids, while a conformational epitope is made up of amino acid residues that are spatially close in the three-dimensional structure of a protein but discontinuous in the primary structure. The impact of heat treatment on linear epitopes is relatively small. Linear epitopes mainly depend on the linear arrangement order of amino acids. Under general heat treatment conditions, the amino acid sequence of proteins does not change. However, if the intensity of heat treatment is too high, it may lead to chemical modifications of amino acids [91]. Conformational epitopes are more susceptible to the influence of heat treatment. This is because heating increases the molecular kinetic energy of proteins and disrupts the weak intermolecular forces within proteins. As a result, proteins undergo denaturation and their conformations change. Conformational epitopes rely on the three-dimensional structure of proteins. Once proteins are denatured, the structure of conformational epitopes is destroyed. No significant difference was observed in the IgG-binding of shrimp tropomyosin upon heating to 80 °C and then cooling to 25 °C [24]. The result suggest that heat-denatured TM retain the allergenicity in the treatment of heating and refold in the process of cooling. Liu et al. reported that the mud crab allergens’ IgE-binding capacity decreased at 115 °C for 15 min [38]. Lasekan et al. found that thermal processing could reduce the allergenicity of TM at 121 °C for 20 min [77]. The binding for Chinese shrimp tropomyosin between allergic peptides and IgE decreased with increasing temperature from 298.15 K to 373.15 K [92]. The effect of heat treatment is related to factors such as heating method and maximum temperature, as well as to factors such as the stability of the allergens and the rupture of chemical bonds. Although traditional thermal technologies were implemented to alleviate shrimp allergenicity, only little research was conducted to evaluate the influence of innovative thermal techniques on modifying shrimp allergens. The main reason might be that the nutrient compositions and organoleptic properties of food material are easily impacted by long-time high-temperatures exposure of thermal techniques. In addition, because of the heat stability of most crustacean allergens, heat treatment is not a preferred method for eliminating crustacean allergenicity.

#### 8.1.2. Microwave

Microwave techniques are electromagnetic waves, which are broadly applied for food processing such as thawing, baking, drying, pasteurization, and sterilization. The most common frequency range of microwaves in the food industry is 300 MHz–300 GHz. Microwaves can change the original structure of proteins, influencing the sensitized ability of certain proteins. In comparison to the traditional heat treatment, microwave techniques exhibit many advantages, such as a more even thermal effect, environment friendliness, lesser energy consumption, high heating rate, and ease of operation. Meanwhile, microwave techniques also exhibit a lesser negative impact on the nutritional compounds and flavor of food products [73].

One study showed that after being processed in a microwave for 15 min at 125 °C, the shrimp TM’s allergenicity was reduced by up to 75%. However, it was found by competitive indirect ELISA analysis that the allergenicity of shrimp was slightly reduced after microwave processing (2450 MHz) at ~17 °C for 1–20 min in the ice water bath. These opposite phenomena might be attributed to the fact that shrimp TM is a heat-stable protein which helps sustain great allergenic potential, particularly with thermal treatment at low temperature. To date, only little research has investigated the microwave treatment to eliminate and modify shrimp allergens [93]. It is urgently needed to develop innovative non-thermal processing techniques.

#### 8.1.3. Irradiation

Irradiation is considered as a useful tool for food preservation and processing with minor modification in the sensory and nutritional features. Irradiation could alter the conformational structure by inducing amino acid modification, cross-linking, aggregation, and fragmentation in food proteins that influence their potential allergenicity. Such shifts might be attributed to free oxygen radicals formed via the radiolysis of water during the irradiation of proteins.

Food irradiation could expose food to ionizing radiation, which leads to shifts in the protein destruction and structure of allergen epitopes. One possible reason is that the irradiation energy is absorbed by allergens. In addition, other reactions like Maillard and glycosylation might happen during the food irradiation processing. The Gamma irradiation technique is widely used to modify the allergenicity of crustacean allergens. It demonstrated that the quantity of tropomyosin, which is the major crustacean allergen, was diminished by Gamma radiation, relying on the dose. Byun et al., 2000 indicated that the main allergen of shrimp can lose its allergenicity under the condition of 10 kGy irradiation. In addition, another research indicated that the shrimp tropomyosin’s IgG binding capacity was significantly reduced by 7 kGy [86]. Rao revealed that γ-ray irradiation below 5 kGy enhanced the allergenicity of shrimp allergens, and irradiation above 10 kGy could reduce allergenicity [68]. Irradiation can expose hydrophobic groups, reduce the thermal stability of proteins, and destroy their allergic epitopes [94]. Based on the above studies, it is worth noting that as a low dose of irradiation could enhance allergenicity, it might not be an ideal to control crustacean allergens.

#### 8.1.4. High Pressure

High-pressure processing, a non-thermal technology, plays an important role in improving product texture through protein alteration and denaturation. It could inhibit the growth of microorganisms in food industries and help to preserve crustacean quality and extend shelf life. High-pressure processing greatly influences the non-covalent bonds of protein molecules such as hydrophobicity, electrostatic interaction, and hydrogen bonding. Therefore, the conformational structure of the protein and its influence on allergenicity might be destroyed by high-pressure technology [95]. Faisal et al. reported that the allergenicity of shrimp allergens was alleviated after household pressure cooking [80]. According to the result reported by Faisal et al. [78], the allergenicity of banana prawn tropomyosin was decreased at 600 MPa for 10 min. Furthermore, with the treatment of 0.14 MPa for 20 min, the decrease allergenicity of *Penaeus monodon* tropomyosin was also found [90]. According to the reported literature, shrimp allergenicity can be effectively reduced by high-pressure processing. Moreover, it also could maintain the original features of foods, like nutritional and organoleptic qualities [76]. In comparison with traditional thermal techniques, high pressure exhibited its advantages of environmental friendliness and low energy consumption. Thus, high-pressure processing displays a potential role to reduce the allergenic proteins of shrimps.

#### 8.1.5. Ultrasound

In the food industry, high-intensity ultrasound emerged, typically applied for dehydration (fruits and vegetables), filtration (fruit juice and dairy whey solutions), homogenization (mayonnaise), and tenderization (meat) processes [96]. High-energy mechanical waves (20–100 kHz) applied by high-intensity ultrasound could induce cyclic generation and collapse of cavities (sonication bubbles) followed by the production of localized regions caused by high temperature and pressure surrounding these collapsed cavities. This leads to conformational shifts in proteins, impacting their potential allergenicity. Dong et al. applied ultrasound treatment (20 kHz, 400 W, 20 min) to purify the shrimp allergen [80]. The result demonstrated that the level of shrimp tropomyosin was reduced by up to 76%, owing to the alterations of protein secondary structure. Li et al. [81] reported that the allergenicity of the shrimp allergen declined by 75% by ultrasound treatment (30 kHz, 800 W, 30–180 min). Zhang et al. indicated that TM’s stability and allergenicity significantly reduced owing to the destruction of TM molecules caused by high-intensity ultrasonic processing (15 min at 20 kHz and 100–800 W) [79]. Ultrasound technology is considered as an effective method for mitigating the shrimp allergen with various advantages, including lowering heating, concentration gradients, higher efficiency, etc. It can affect the structure of food components and then improve the physical, chemical, and functional properties of food [97].

#### 8.1.6. Cold Plasma

Cold plasma, the fourth state of gas and matter, is composed of active particles like ions, free radicals, excited atoms, molecules, and electrons, generating active oxidation system waves of reactive oxygen species (ROS) and reactive nitrogen species. In general, because of the low temperature and energy level of cold plasma, it is used as an innovative tool for altering the conformation and functions of food production [83]. These shifts generally contain the protein–protein cross-linkages and cleavage of peptide bonds, resulting in the changes in protein structures. It is currently used in food packaging material and changing food properties, etc. Ekezie [82] demonstrated that 76% of the allergenicity of shrimp TM decreased under direct dielectric discharge plasma, which might be responsible for the change in shrimp epitopes. In addition, one study showed that after the plasma exposure for 15 min, the binding capacities of IgE and IgG were maximally reduced by 17.6% and 26.87%, respectively [98]. More studies are still being explored to control crustacean allergy by cold plasma.

#### 8.1.7. Pulsed Light

Pulsed light (PL) consists of significantly high- and low-power pulses of white light with a broad spectrum [84]. The light spectrum includes ultraviolet light (54%), visible light (26%), and infrared light (20%). Proteins contain efficient chromophores, which could cause side-chain oxidation, backbone fragmentation, and protein cross-linking and aggregation by absorption of light. The PL treatment might alter the allergens’ IgE binding epitopes. Moreover, because of the loss of conformational epitopes, the shrimp allergen conformation could be changed by PL light. For instance, the IgE binding capacity of shrimp allergens could be dramatically diminished by the treatment of PL (3 pulses/s rates, 360 μs width, 10 cm distance from the light source) [99]. In addition, the IgE-binding capacity of tropomyosin in shrimp extracts is reduced with the treatment of PL (360 μs for 4 min, 3 pulses/s) [82]. The reported research provided good prospects for reducing shrimp allergenicity. More studies are needed to apply PL for processing the shrimp allergen.

### 8.2. Chemical Methods

#### 8.2.1. Maillard Reaction

During thermal processing, the Maillard reaction is a well-known non-enzymatic interaction between sugars and proteins. The Maillard reaction refers to the amino group on the protein molecule, mainly the ε- of lysine (Lys) amino groups or carboxyl groups of carbohydrates, and forms covalent bonds combined with a chemical reaction [85]. In food products, the type and level of Maillard reaction depend on diverse variables, covering reducing sugars, the ratio of amino groups, the structures of (poly)saccharides and proteins, time, and temperature. Some studies have reported the influence of glycation on the allergenicity of TM. Zhang et al. [46] revealed that the IgG/IgE binding capacity of shrimp TM was reduced following glucosamine-catalyzed glycation. The allergenicity of shrimp TM was decreased by a Maillard reaction with galacto-oligosaccharide, chitosan-oligosaccharide, or ribose [86].

#### 8.2.2. Acid Treatment

The study on crustacean allergens after acidic treatment is scant. Ekezie [82] elucidated that acidic conditions could cause the allergenicity changes in shrimp TM. White vinegar (at different pH levels of 1.0, 2.5, 3.5, and 4.8) was used to evaluate the impact of acid treatment on TM allergenicity. Compared to samples marinated at pH 4.8 and control, the IgE binding capacity of TM was significantly reduced below pH 3.5.

### 8.3. Biological Methods

#### 8.3.1. Microbial Fermentation

In the food industry, fermentation displays an essential role in strengthening food quality and microbiological stability. It occurs when microorganisms break down food components, like converting sugars to organic acids, alcohol, and carbon dioxide without oxygen. Fermentation is essentially related to microorganisms and environmental factors, such as substrate concentration, pH, and temperature. Interestingly, fermentation could break down proteins into small molecular peptides and amino acids. It could also keep the nutrients of the food and help absorption. Typically, specific characteristic requirements determine the selection of fermentation microorganisms, like Saccharomyces cerevisiae in alcoholic beverages, Lactobacillus (lactic acid bacteria) in yogurt, Pediococcus pentosaceus in sausages, and molds in soy-based products (e.g., tempeh, shoyu, and miso). Moreover, fermentation can also be utilized for modifying the conformational structure of allergenic proteins.

Park et al. [86] found that the IgG binding capacity of shrimp allergens was reduced by 95% after fermentation with 10% salt at 25 °C for 5 days. The IgG binding capacity was significantly reduced for high-temperature and low-salt conditions. It was found that the IgE binding capacity of shrimp TM could be reduced by suitable temperature (25 °C), low salt level (10%), and longer fermentation periods owing to protein hydrolysis [86]. This result suggests that shrimp allergenicity can be reduced by fermentation under the conditions of long time durations and relatively suitable temperatures [86]. Notably, in comparison with other technologies, food allergenicity could be significantly diminished by fermentation due to its low cost and safety [13].

#### 8.3.2. Genetic Engineering

Genetic engineering could be directly applied to food allergens. The new amino acid sequences are produced, and the expression of allergic epitopes is blocked through gene recombination, knockout, and mutation of specific genes. In [87], it was found that the allergenicity of a new protein VR9–1 can be reduced by 98% when shrimp allergens induced mutations. However, as a product of genetic engineering technology, genetically modified organisms (GMOs) are not accepted in the food system in many parts of the world, because genetic modification operations may introduce new allergens into food.

### 8.4. The Trend of Combining Multiple Methods to Reduce the Allergenicity of Crustaceans

The combination of multiple methods appears to be a new trend in decreasing crustacean allergenicity. It has been shown that the IgE binding capacity of shrimp samples could be significantly reduced by galactose treatment with high-temperature pressure (0.08 MPa, 115 °C, 6 min) [60]. Li et al. indicated that the immunoreactivity of shrimp muscles could be considerably decreased by Gamma radiation with boiling treatment [67]. Hu et al., 2019 found that the immunoreactivity of shrimp tissue appeared be much lower with the synergy of enzymatic hydrolysis and a high pressure of 450 MPa (40 °C, 55 min) [100]. Wang et al., 2023 showed that the integration of glycation treatment (80°C, 4 h) and CP (dielectric barrier discharge, 1.0 A, 60 kV) could decrease up to 40% of IgE the binding capacity of TM [88]. The combination of multiple methods could lead to significant influence on decline in crustacean allergenicity, which may also help maintaining and even enhancing food quality. However, limited studies have reported on the hurdle treatment of crustacean samples. Further studies are needed to achieve the best method for decreasing crustacean allergenicity.

## 9. Strategies to Address the Risk of Crustacean Allergens

### 9.1. Avoidance and Labeling

At present, there is no effective prevention and eradication method for food allergy in clinical practice. The current effective way to prevent crustacean allergy is to avoid the consumption of crustacean foods. However, most foods that contain diverse allergenic components are the essential source of human nutrition, and many allergenic components cannot be seen in their original state. Therefore, allergen labeling should be present on food packaging. Furthermore, many countries have built specific legislation regarding the mandatory labeling of allergenic foods to protect public health [100]. The allergenic food list might vary with specific country legislation; crustaceans are normally covered. Moreover, education on food allergy should be strengthened in order to improve public awareness for food allergy protection.

### 9.2. Immunotherapy

Understanding the mechanism of food allergy could help develop alternative ways of treatment to minimize sensitivity and stimulate tolerance to the dietary protein. According to the administration route of allergens, immunotherapy techniques can be classified as sublingual, epicutaneous, and oral immunotherapies. All these immunotherapy methods are in the research phase, and other immunotherapy methods aimed at improving the allergic patient’s life are currently in various stages of clinical trials. They minimize the IgE binding potential of the allergen via food processing approaches. Rat basophil mediator release assays confirm that mutant TM from crustaceans is developed with decreased allergenic potential, and significantly declined activation was observed in a murine model of food allergy [13]. Such novel hypoallergenic proteins are a safer way of protection against crustacean allergy.

## 10. Conclusions and Prospect

Crustacean allergy is considered an important public safety issue. The study focused on collecting basic information about the different characteristics, structures, and functions of crustacean allergens. The different evaluation and detection methods were discussed. A technical platform was constructed by a combination of different evaluation methods to evaluate crustacean allergenicity better. Moreover, sensitive, selective, reliable, rapid, and cost-effective analytical tools are needed to detect crustacean allergens in food processing. In addition, the combination of multiple methods could lead to better effect in decreasing crustacean allergenicity. For example, the combination of enzymatic hydrolysis and heat treatment was shown to significantly alter the allergenic epitopes of crustacean proteins. Enzymatic hydrolysis breaks down the protein into smaller fragments, while heat treatment further modifies the conformation of these fragments, reducing their ability to bind to IgE antibodies. The review also provided some strategies to address the risk of crustacean allergy, which are significant and much needed to assist in understanding and controlling food allergens in future studies. In-depth research is still needed to determine new crustacean allergens and broaden the awareness of existing allergens. The safety and health of allergy patients can be protected to a maximized extent through a comprehensive understanding of allergens.

## Figures and Tables

**Table 1:** Basic information on molecular weight and biological functions of crustacean allergens.

Biochemical Names	Molecular Weight	Heat Resistance	Biological Function	Reference
Tropomyosin	34–38 kDa	Yes	Structural protein	[13]
Arginine kinase	40 kDa	No	Energy metabolism	[11]
Sarcoplasmic calcium-binding protein	20–22 kDa	Yes	Calcium ion buffering	[14,15]
Myosin light chain 1	17–23 kDa	Yes	Structural protein	[15]
Myosin light chain 2	17–23 kDa	Yes	Structural protein	[16]
Troponin C	21 kDa	-	Structural protein	[15,17]
Hemocyanin	75 kDa	Yes	Oxygen transport	[18]
Paramyosin	100 kDa	No	Muscle contraction	[19]
Aldolase A	∼40 kDa	-	Enzyme in glycolysis	[20]
Myosin heavy chain	225 kDa	-	Muscle contraction	[17]
a-Actin	31–42kDa	-	Muscle contraction	[21]
Triosephosphate isomerase	28 kDa	-	Glycolytic enzyme	[15,20]
smooth endoplasmic reticulum Ca^2+^ ATPase	113	-	Enzyme	[21]
glyceraldehyde-3-phosphate dehydrogenase	37	-	Enzyme	[20]

Note: The symbol “-” under “Heat Resistance” indicates that the information was not provided in the original study.

**Table 2:** Recent advances in physical, chemical, and biological methods on crustacean allergens.

Reduce Method		Food Material	Parameters	Allergenicity	Reference
Physical method	Heat	Shrimp TM	Heat to 80 °C and then cool to 25 °C	A little decreased	[23]
		Mud crab TM	115 °C, 15 min	Retains IgE binding capacity	[35]
		shrimp TM	T = 298.15 K–373.15 K	Decreases the IgE binding capacity	[46]
		Shrimp powder	75–125 °C for 5–15 min	Reduced by up to 75%	[53]
		Brown shrimp	0, 1, 3, 5, 7, or 10 kGy	Reduces the binding ability of immunoglobulin E	[55]
		Shrimp TM	0, 1, 3, 5, 7, 9 kGy	Decrease in IgG binding capacity at 7 kGy	[73]
		Shrimp TM	below 5 kGy, above 10 kGy	Enhanced below 5 kGy; reduced above 10 kGy	[67]
	High pressure	Shrimp extracts	0.14 MPa, 121 °C, 20 min	Reduced (weaker tropomyosin band)	[77]
		Shrimp muscle	House-hold pressure cooker, 5–20 min	Tropomyosin band retained	[39]
		Shrimp TM	600 MPa, 5 and 10 min, 120 °C	Decreasing (weaker tropomyosin band)	[78]
	Ultrasound	Shrimp TM	20 kHz, 100–800 W, 15 min	Reduced (α-helix content reduced)	[79]
		Shrimp TM	20 kHz, 400 W, 20 min	Reduced by up to 76%	[80]
		Shrimp muscle	30 kHz, 800 W, 30–180 min	Declined by 75%	[81]
	Cold plasma	Shrimp TM	Cold argon plasma jet, 15 min	Reducing IgE (17.6%) and IgG (26.8%) binding capacities	[82]
		Shrimp TM	30 kV, 60 Hz, and 5 min	Reduced by up to 76%	[83]
	Pulsed light	shrimp extracts	3 pulses/s, 360 ms	Decreased the IgE binding capacity	[84]
		Shrimp extracts	3 pulses/s, 360 ms, 4 min	Reduced by 24% for IgE binding capacity	[83]
Chemical method	Maillard reaction	Shrimp TM	Ribose, galacto-oligosaccharide, chitosan-oligosaccharide	Reduced by up to 60%	[85]
		Shrimp TM	glucose, maltose, and maltotriose	Reduced the IgG/IgE binding (the secondary and tertiary structures changes)	[44]
	Acid treatment	shrimp TM	pH (1.0, 2.5, 3.5, and 4.8)	Reduced allergenicity	[77]
Biological method	Fermentation	Shrimp TM	10% salt at 25 °C, 5 days	Reduced the IgG binding capacity by 95%	[86]
		Salt-fermented shrimps	10–25% of salt concentrations at 5–25 °C, 1 year	Significantly reduced (binding ability decreased to 5%)	[86]
	Genetic engineering	Shrimp TM	-	Reduced by 90–98%	[13]
Combined treatment methods	Acetic acid with pressure	Soaked shrimp	5% acetic acid with pressure cooking (using house-hold pressure cooker), 0–30 min	Reduced (faint or absent tropomyosin band)	[39]
	High-temperature pressure with Galactose treatment	Shrimp muscle	0.08 MPa, 115 °C, 6 min	Reduced (weaker tropomyosin band)	[54]
	High pressure with enzymatic hydrolysis	Shrimp muscle	450 MPa, 40 °C, 55 min	Reduced immunoreactivity	[14]
	Hydrolysis with thermal treatment and high pressure	Shrimp TM	30 min, 3000 U/g of trypsin, 40 °C, normal pressure or 200 MPa	Reduced by 89% or 98%	[87]
	Cold plasma and glycation treatment	Shrimp TM	CP (dielectric barrier discharge, 60 kV, 1.0 A) combined with glycation treatment (4 h, 80 °C)	Reduced allergenicity	[88]

## Data Availability

No new data were created or analyzed in this study. Data sharing is not applicable to this article.
